# MicroRNA in Human Glioma

**DOI:** 10.3390/cancers5041306

**Published:** 2013-10-23

**Authors:** Mengfeng Li, Jun Li, Lei Liu, Wei Li, Yi Yang, Jie Yuan

**Affiliations:** 1 Key Laboratory of Tropical Disease Control (Sun Yat-sen University), Chinese Ministry of Education, Guangzhou 510080, China; E-Mails: lijun37@mail.sysu.edu.cn (J.L.); liulei3@mail2.sysu.edu.cn (L.L.); weili6363@gmail.com (W.L.); yangyi23@mail.sysu.edu.cn (Y.Y.); yuanjie@mail.sysu.edu.cn (J.Y.); 2 Department of Microbiology, Zhongshan School of Medicine, Sun Yat-sen University, Guangzhou 510080, China; 3 Department of Biochemistry, Zhongshan School of Medicine, Sun Yat-sen University, Guangzhou 510080, China; 4 Department of Pharmacology, Zhongshan School of Medicine, Sun Yat-sen University, Guangzhou 510080, China; 5 Key Laboratory of Functional Molecules from Oceanic Microorganisms (Sun Yat-sen University), Department of Education of Guangdong Province, Guangzhou 510080, China

**Keywords:** glioma, microRNA, biological function, gap filler, amplifier, crosstalk, biomarker

## Abstract

Glioma represents a serious health problem worldwide. Despite advances in surgery, radiotherapy, chemotherapy, and targeting therapy, the disease remains one of the most lethal malignancies in humans, and new approaches to improvement of the efficacy of anti-glioma treatments are urgently needed. Thus, new therapeutic targets and tools should be developed based on a better understanding of the molecular pathogenesis of glioma. In this context, microRNAs (miRNAs), a class of small, non-coding RNAs, play a pivotal role in the development of the malignant phenotype of glioma cells, including cell survival, proliferation, differentiation, tumor angiogenesis, and stem cell generation. This review will discuss the biological functions of miRNAs in human glioma and their implications in improving clinical diagnosis, prediction of prognosis, and anti-glioma therapy.

## 1. Introduction

Annually, 3.5 in 100,000 people are diagnosed with central nervous system (CNS) cancer, accounting for 1.9% of all new cancer cases and 2.3% of cancer deaths worldwide [1]. Glioma, the most common neuroepithelial cancer, represents the majority of malignant brain tumors in humans [2]. Gliomas are clinicopathologically categorized into grades I–IV according to the World Health Organization (WHO) grading criteria [3]. Among all glioma cases diagnosed, a majority belongs to grade IV, also known as high-grade astrocytoma or glioblastoma (GBM) [2,4]; despite aggressive therapies, over 50% of patients die within one year of diagnosis [5,6]. Current standard therapies for newly diagnosed glioma includes surgical resection followed by adjuvant radiotherapy and/or chemotherapy. Nevertheless, surgical resection is often limited by a lack of clear primary tumor margin and by their locations at vital anatomical structures. Moreover, radiotherapy and chemotherapy effectiveness is suboptimal in many cases, largely due to readily developed resistance to radiation and chemotherapeutic drugs [7]. A thorough understanding of the molecular mechanisms underlying glioma development and progression and its radioresistance and chemoresistance is crucial for improving conventional therapeutic strategies and developing novel therapies against the disease.

Numerous signaling pathways and molecules are involved in glioma pathogenesis. Genetic alterations and/or deregulated expression of genes and their protein products have been identified in gliomas. Amplification and/or mutation of the epidermal growth factor receptor (EGFR) gene were found in up to 60% of primary GBM cases [8] and are believed to be associated with the development of the malignant phenotype of glioma cells, e.g., proliferation and invasiveness. Furthermore, in nearly half of anaplastic astrocytoma and almost all glioblastoma cases, the p16-cyclin-dependent kinase (CDK)4/CDK6-cyclin D1 (CCND1)/retinoblastoma (RB) pathways [9] that negatively regulate cell cycle progression are aberrant, leading to uncontrolled tumor cell proliferation. Another important growth factor, platelet-derived growth factor (PDGF), and its receptor are upregulated in human glioma and are involved in the development of the malignant phenotype, including tumor angiogenesis [10]. Other molecular changes in the pathological process of glioma include deregulated expression or dysfunction of tumor protein 53 (TP53) and phosphatase and tensin homolog (PTEN) [11,12,13]. Despite the accumulated findings on the molecular changes, unidentified mechanisms underlying the pathogenesis of glioma remain to be understood. To understand the molecular network involved in initiating or promoting glioma development and progression, the gaps between known signaling events within a mechanistic cascade must be filled and the molecules that mediate crosstalk among different signaling pathways identified. Furthermore, it remains puzzling how certain self-regulated feedback loops, which would otherwise be under tightly regulated control, are disrupted and thereby become oncogenic. Clarification of these issues is urgent and key to complete understanding of glioma and to the development of effective intervention strategies.

Research in the past decade has brought microRNAs (miRNAs) to the forefront of cancer molecular biology. Experimental and clinical scenarios have widely shown that miRNAs play pivotal roles in human cancer development and progression. MiRNAs, a class of non-coding 20–22-nucleotide RNAs, are encoded in introns, exons, or the intergenic regions, and miRNA-coding genes usually locate at fragile sites of chromosomes [14,15]. MiRNAs regulate gene expression via imperfect and sometimes perfect base pairing with specific sequences, leading to altered transcription and/or translation. In most cases identified thus far, miRNAs silence gene expression by suppressing mRNA translation through sequence-specific hybrids between the miRNA 5' proximal “seed” region (position 2–8) and the 3' untranslated region of mRNAs, leading to mRNA destabilization or degradation [16,17,18,19,20]. Based on such molecular interactions, the modulatory effect of a particular miRNA usually exhibits a multi-target property, providing a new dimension of regulation for gene expression and usually consequently altering the quality and/or quantity of molecular signaling in a biological context with high efficiency. Indeed, such gene expression modifications by miRNA-mediated changes are significant not only in normal physiological processes, but also in pathological conditions, including various types of cancer, for example, glioma.

## 2. miRNA Biological Functions in Glioma

Glioma is the cancer type with which the functional significance of miRNAs in human cancer was discovered. Since the early studies that identified in human glioma the importance of miRNAs, including miR-21 [21,22], a significant number of miRNAs have been found to be deregulated in glioma and contribute to disease development and progression. Similar to those found in other cancers, abnormalities in miRNA expression or function in glioma are usually associated with the development of representative biological hallmarks that distinguish malignant cells from normal ones, including increased cell proliferation, abrogated apoptosis, enhanced invasiveness and cell motility, and promotion of angiogenesis. With these phenotypic changes, the identified miRNAs target common oncogenic or tumor-suppressive pathway components and are therefore believed to be mechanistically linked to the role of miRNAs in glioma. Table 1 summarizes the miRNAs found in experimental or clinical human gliomas, and some of their postulated functions are discussed below.

### 2.1. miRNAs Modulate Glioma Cell Proliferation

Accelerated proliferation is a cancer cell characteristic that contributes to the uncontrolled growth of a malignant tumor at almost all stages of disease progression. This biological feature is believed to be particularly important in glioma, as the fast growth of this brain tumor is key to its high lethality [4]. In this context, while some genetic alterations in cell cycle regulators, e.g., loss of cyclin-dependent kinase inhibitor 2A (CDKN2A) or RB function and CDK4 amplification, are associated with promoting glioma cell proliferation [9], changes in miRNA species modulate key nodes to similarly alter proliferative signaling pathways. For example, miR-137 is downregulated in grade III and IV human gliomas. As miR-137 targets and suppresses CDK6 expression, a positive mediator of cell cycle progression, its downregulation enhances glioma cell proliferation, and lower miR-137 levels indicate a poorer prognosis [23,24]. MiR-34a, the expression of which is diminished in glioma cells, targets the mRNAs of multiple growth-promoting genes, including E2F transcription factor 1 (E2F1), hepatocyte growth factor receptor (c-met), and CCND1. Expression of miR-34a reverses the ability of these oncoproteins to sustain the growth of glioma cells *in vitro* and glioma xenografts *in vivo* [25,26,27].

**Table 1:** MiRNA biological functions in glioma.

MiRNA	Target genes	Biological effects ^a^	Postulated upstream regulators ^b^	References
Mir-7	EGFR, IRS-2, FAK	Invasion (−), Radioresistance (−), Migration (−)		[28,29,30,31]
MiR-10b	HOXD10, Bim, TFAP2C, p16, p21, CSMD1	Invasion (+), Proliferation (+), Cell cycle (+), Apoptosis (−), Autophagy (−)		[32,33,34,35]
Mir-15b	CCNE1, NRP2	Cell cycle (−), Invasion (−), Angiogenesis (−)		[36,37]
Mir-16-1	Zyxin	Migration (−), Invasion (−), Proliferation (−)		[38]
Mir-17–92 cluster	CDKN1A, E2F1, Pold2, PTEN, CTGF	Proliferation (+), Apoptosis (−), Differentiation (−)	c-Myc (+)	[39,40,41,42]
Mir-21	RECK, pdcd4, CDC25A, HNRPK, TIMP-3, TAp63, LRRFIP1	Invasion (+), Migration (+), Proliferation (+), Apoptosis (−), Autophagy (−), Resistance to TMZ, taxol and VM-26 (+), Radioresistance (+)	β-catenin/STAT3 pathway (+)	[43,44,45,46,47,48,49,50,51,52,53,54,55,56,57,58]
Mir-23b	VHL,Pyk2, HMGA2	Proliferation (±), Invasion (±), Apoptosis(−), Migration(−), Cell cycle(−)		[59,60,61]
Mir-26a	PTEN			[62]
Mir-30e*	IκBα	Invasion(+), Angiogenesis(+)		[63]
Mir-31	Radixin	Migration(−), Invasion(−)		[64]
Mir-34a	E2F1, Notch1, Notch2,C-met	Proliferation(−), Apoptosis(+),	P53	[25,65,66]
Mir-101	EZH2	Proliferation(−), Migration(−), Angiogenesis(−),		[67]
Mir-124	SNAI2	Stemness(−), Invasion(−)		[68]
Mir-125b	BMF, E2F2	Angiogenesis(−), Proliferation(±), Apoptosis (−)	VEGF (−)	[69,70,71,72]
MiR-125b-2		TMZ resistance(+)		[73]
Mir-128	Bmi-1, E2F3a, p70s6k1, RTKs	Proliferation(−), Self-renew(−), Apoptosis(+), Angiogenesis(−), Differentiation(+)	Ginsenoside Rh2 (+)	[74,75,76,77,78]
Mir-137	Cox-2, CDK6	Iinvasion(−), Migration(−), Proliferation(−), Stmeness(−), Cell cycle(−)		[23,24]
Mir-145	CTGF, ADAM17, NEDD9	Invasion(−), Migration (−), Proliferation(−), Stmeness(−)		[79,80,81,82]
Mir-146	EGFR	Invasion(−), Migration(−)		[83]
Mir-152	MMP-3	Invasion(−), Angiogenesis(−)		[36]
Mir-153	IRS-2, Mcl-1, Bcl-2	Apoptosis(+), Proliferation(−)	Chromatin-modifying drugs (+)	[84,85]
Mir-181	Bcl-2	Apoptosis(+), Invasion(−), Proliferation(−), Radiorasistance(+)		[86,87]
Mir-182	CYLD	Proliferation(+), Angiogenesis(+)	TGF-β (+)	[88]
Mir-184	Npm1, Akt2	Proliferation(−), Invasion(−)		[89]
Mir-195	E2F3, CCND3, Cyclin D1, Cyclin E1	Proliferation(−), Invasion(−), TMZ resistance(+)		[90,91,92]
Mir-204	SOX4, EphB2	Self-renewal(−), Stemness(−), Migration(−), Invasion(−)	Methylation (+)	[93]
Mir-221/222	TIMP3, PUMA, p57, PTEN, p27, ICAM-1, CX43, PTPμ	Invasion(+), Proliferation(+), Apoptosis(−), Radioresistance(+), TMZ resistance (+), Cytolysis to CTL (−)	NF-κB (+), C-Jun (+)	[94,95,96,97,98,99,100,101,102,103]
Mir-296	HGS	Angiogenesis(+)	VEGF (+), EGF (+)	[104]
Mir-326	PKM2, NOTCH1, NOTCH2	Proliferation(−), Apoptosis(+), Invasion(−)	Notch (−)	[105,106,107]
Mir-410	MET	Proliferation(−), Invasion(−)	Methylation (−)	[108]
Mir-451	CAB39	Response to metabolic stress	Glucose (+)	[109]
Mir-455		TMZ resistance(+)		[90]
Mir-486	CYLD, ITCH, TNIP-1, TNIP-2, TNIP-3	Aggressiveness(+), Angiogenesis(+), Proliferation(+), Invasion(+)		[110]
Let-7		Migration(−), Proliferation(−)		[111]

**^a^** (+) = promoted, (−) = inhibited; **^b^** (+) = upregulated, (−) = downregulated.

In contrast, some miRNAs can induce excessive proliferative signaling. The upregulation of an oncogenic miRNA (oncomir) promotes glioma cell proliferation. MiR-182 can suppress cylindromatosis (CYLD), a deubiquitinase that mediates ubiquitin deconjugation and acts as an inhibitor of nuclear factor κB (NF-κB) activation, leading to overactivation of NF-κB signaling. MiR-182 is transcriptionally upregulated by transforming growth factor β (TGF-β) in glioma tissues and predicts poor prognosis in glioma [88].

### 2.2. miRNAs Modulate Glioma Cell Apoptosis

Almost all cancer types feature suppression of apoptosis. In glioma, it has been extensively shown that the anti-apoptotic capability of tumor cells is associated with disease progression and resistance to therapies. In a normal cell population, the balance between apoptosis and survival is well maintained and controlled by a network of pro-apoptotic and anti-apoptotic regulators; this elegantly formed modulatory network is disrupted in almost all cancers, usually characterized by pro-apoptotic factor suppression and/or anti-apoptotic (or survival) factor overactivation [112]. Recent studies have established that alterations in miRNA expression or function are often involved in the dysregulation of cell death during human glioma development and progression. For example, miR-181 and miR-153 promote apoptosis by directly targeting B-cell chronic lymphocytic leukemia/lymphoma 2 (Bcl-2) mRNA and repressing its translation, thereby inhibiting gliomagenesis [84]. Both miR-181 and miR-153 expression is decreased in glioma cell lines and a subset of clinical glioma specimens, suggesting roles of the two miRNAs in glioma progression. Moreover, miR-181 downregulation is more prominent in grade III and IV glioma than that in lower grades [84,87].

Conversely, upregulation of certain miRNA also inhibits apoptosis in glioma cells and supports cell survival. For example, miR-26a that is upregulated in glioma tissues suppresses PTEN, RB1 and MAP3K2/MEKK2 expression and inhibits c-JUNN-terminal kinase-dependent apoptosis [62,113]. Other anti-apoptotic miRNAs found in glioma cells are miR-221/222 and miR-21, which are both upregulated and indicated as oncogenic in glioma [43,94].

### 2.3. miRNAs Modulate Glioma Cell Invasion and Migration

The high lethality of glioma, particularly GBM, is closely associated with its ability to infiltrate the surrounding tissue, resulting in both the destruction of normal brain regions and a lack of clear border around the malignant lesion, which leads to rapid post-surgery recurrence due to incomplete resection. It is recognized that this biological feature can be acquired early in tumorigenesis [9]. Molecular mechanisms underlying glioma cell invasion and migration usually involve factors that promote degradation of the extracellular matrix (ECM) or cell motility. Recent studies have identified miRNAs contributing to the above processes, and are thereby indicative of disease aggressiveness.

MiR-30e*, which represents the product of complementary DNA strand coding for miR-30e, is involved in promoting glioma cell invasiveness. By activating NF-κB inhibitor (IκBα)/NF-κB signaling, miR-30e* triggers the overexpression of invasion-promoting factors, mainly including matrix metalloproteinase-9 (MMP-9), an important enzyme that degrades ECM [63]. Moreover, miR-21 can downregulate tissue inhibitor of MMPs-3 (TIMP3) and reversion-inducing cysteine-rich protein with Kazal motifs (RECK), which are both MMP-9 inhibitors, resulting in elevated MMP-9 activation and enhanced tumor cell invasion [44]. *In vitro* invasion assays and orthotopic xenograft models, both miR-30e* and miR-21 are upregulated in glioma cells and promote their invasiveness by mediating ECM degradation. In the clinic, the two miRNAs correlate with malignant progression and poor survival [21,45,63].

MiRNAs that antagonize glioma cell invasion have also been identified. MiR-218 can directly target lymphoid enhancer-binding factor 1 (LEF1) and downregulate the expression of its downstream proteins, e.g., MMPs, reducing ECM degradation, whereas inhibition of miR-218 expression enhances glioma cell invasiveness, suggesting a tumor-suppressive role of the miRNA. MiR-218 is expressed at low levels in glioma tissues, particularly in GBM, which might contribute to the acquisition of invasive potential [114].

### 2.4. miRNAs Modulate Glioma Angiogenesis

GBM is one of the most highly vascularized human cancers. It is well recognized that the progression of human glioma is angiogenesis-dependent. Indeed, glioma is one of the first cancers in which angiogenesis was found to be a key phenotypic feature to disease progression [115]. Accumulated evidence has shown that high vascular density, glomeruloid vessels in particular, and pro-angiogenic factor upregulation are indicative of poor prognosis for patients with gliomas [9]. Aberrant activation of pro-angiogenic signaling pathways that are common in human cancers, e.g., the EGFR/phosphatidylinositol-3-kinase (PI3K)/Akt, vascular endothelial growth factor (VEGF), PDGF, and NF-κB pathways, is important in the development of neovessels in human gliomas [9,115,116,117,118]. Recently, miRNAs whose upregulation promotes angiogenesis, or whose downregulation suppresses tumor vasculature, were found to play a role in modulating the angiogenic properties of human glioma.

In addition to miR-30e* and miR-182, which activate NF-κB signaling and subsequent expression of downstream pro-angiogenic factors such as VEGF-C and MMPs [63,88], other miRNAs have been identified as stimulators of angiogenic endothelial proliferation, invasion, adhesion, or migration. MiR-296 levels are elevated in primary human brain microvascular endothelial cells, likely by glioma cell-derived vascular growth factors, and act as a promoter of angiogenesis by targeting hepatocyte growth factor-regulated tyrosine kinase substrate, a protein mediating the degradation of the growth factor receptors VEGFR2 and PDGFRβ [104].

In contrast, miR-15b attenuates the expression of a VEGF receptor, neuropilin-2 (NRP-2), abrogating VEGF-induced angiogenesis. miR-15b downregulation in glioma cells is associated with progression of glioma angiogenesis [36]. Similarly, miR-128 inhibits P70s6 kinase 1 (P70s6k1) translation and suppresses the expression of its downstream effectors, hypoxia-inducible factor 1 (HIF-1) and VEGF, which are both key mediators of tumor angiogenesis, reducing vascularization. MiR-128 levels are lower in glioma than in normal brain tissue and are associated with glioma aggressiveness [74,75,76].

### 2.5. miRNAs Are Involved in Glioma Stem Cell Development and Maintenance

Cancer stem cells (CSCs), defined as a tumor cell sub-population capable of self-renewal and heterogeneous cancer cell propagation, [119] are believed to be a pool of cells to which uncontrolled proliferation, metastasis, and resistance to anti-cancer therapies are largely attributed. Glioma was one of the first solid tumor types from which CSCs were identified [120]. Cascades triggered or mediated by signaling pathways such as the Wnt/β-catenin, TGF-β, EGFR, and Notch pathways have been found to play important roles in glioma stem cell (GSC) development and/or maintenance of stemness [121,122,123,124]. Along with these proteins, miRNAs are also reported to play a role in stemness.

Interestingly, miR-204 is markedly downregulated in both GSC and neural stem cells. Sex-determining region Y-box 4 (SOX4), a stemness-governing transcriptional factor, is a miR-204 target. Restoring miR-204 expression in glioma cells restrains the development of the stem cell-like phenotype of glioma cells [93], suggesting that miR-204 plays a role in the development or maintenance of GSC stemness. Moreover, there is increased miR-17–92 locus expression in a subset of GBM cells. By targeting differentiation-promoting genes and cell cycle promoter genes, miR-17–92 inhibits apoptosis and induces cell proliferation in GBM spheroids, in which glioma-initiating cells and GSCs are enriched. Consistently, suppression of miR-17–92 expression by nucleic acid inhibitors or differentiation-inducing agents, e.g., all-trans retinoic acid, reverses the miR-17–92 effects on GBM spheroids, suggesting a key role of miR-17–92 in sustaining GSC stemness and the potential usefulness of miRNAs as targets of GSC-targeting strategies [39].

### 2.6. miRNAs Contribute to Glioma Resistance to Therapies

Radiotherapy and chemotherapy represent the two main conventional anti-glioma treatment strategies. Their effectiveness and efficacy remain limited largely due to the development of radioresistance and chemoresistance in glioma cells and their microenvironment [125]. Relapse following failure of conventional therapies is the most common cause of death for most glioma patients [126,127,128]. Thus, increasing sensitivity to radiation and chemotherapeutic agents and reducing resistance are of great clinical importance. Biologically, there is evidence that resistance to anti-cancer therapies is closely associated with sustained overactivation of anti-apoptotic signaling pathways involved in enhancing and sustaining cell survival [7]. Shi *et al*. reported that miR-21 overexpression mediates resistance to temozolomide, a commonly used alkylating therapeutic agent, by decreasing the Bcl-2-associated X protein (Bax)/Bcl-2 ratio and caspase-3 activity in glioma cells [46]. Moreover, miRNA suppression of leucine-rich repeat (in Flightless I)-interacting protein-1 (LRRFIP1), an NF-κB pathway inhibitor and direct target gene of miR-21, increases the proportion of teniposide-resistant cells [47]. MiR-181a also downregulates Bcl-2 and significantly sensitizes malignant glioma cells to radiation treatment [87]. These findings suggest that miRNAs might be possible targets for overcoming resistance.

GSCs have proven to be highly resistant to radiation and chemotherapy [7,129]. Hence, a better understanding of the molecular mechanisms responsible for sustaining GSC stemness is important for developing new therapeutic strategies to improve the effectiveness of conventional treatments [129]. Therefore, identification of the miRNAs, and their alterations, that contribute to supporting glioma cell survival and stemness is essential for overcoming glioma resistance to treatments.Moreover, other possible mechanisms through which miRNAs can confer resistance to various therapeutic modalities, e.g., modulation of drug pumps, may also exist. Indeed, such miRNAs have been targeted to improve sensitivity to experimental anti-glioma chemotherapy. For example, in the presence of the cytotoxic agent VM-26, miR-21 sustains constitutive NF-κB activation by targeting LRRFIP1, reducing the killing effect of VM-26. In U373MG glioma cells, miR-21 suppression by specific antisense oligonucleotides enhanced VM-26 cytotoxicity [47]. Moreover, miR-195, miR-455-3p, and miR-10a* were shown to mediate glioma cell resistance to temozolomide, although the specific underlying mechanism remains unclear [90].

Studies on the modulatory effects of aberrantly expressed miRNAs on glioma cell biological functions have opened up a new avenue in the understanding of glioma pathogenesis and the development of novel strategies against glioma. Depending on the specific modes of action of these miRNAs in glioma cells and through the upregulation or downregulation of these miRNAs, these molecules act either as oncogenes (oncomirs) or tumor suppressors. It is notable that miRNA post-transcriptional regulation can occur rapidly, with a short response delay, producing a rapid mode of phenotypic changes [130]. Furthermore, due to the sequence-specific but imperfect matching property of a miRNA when interacting with its mRNA target, the modulatory functions of miRNAs exhibit a remarkable multi-target trait, enabling efficient regulation of the gene expression profiles of a cell. These features render the category of molecular regulators an interesting and attractive class of therapeutic targets for complex diseases such as human glioma.

## 3. Biological Importance of miRNAs in Glioma

### 3.1. miRNAs Act as “Gap Filler” in Cellular Signaling Networks

The discovery of miRNAs, and studies on their biological functions and roles in human glioma, provides important new insights in our current understanding of the molecular mechanisms underlying glioma development and progression. The molecules and pathways that contribute to the malignancy of glioma have been identified. However, gaps remain along and between the signaling cascades composed of the identified functional molecular events, and numerous miRNAs have recently been indicated as good fits as “fillers” of such gaps. For example, cyclic adenosine monophosphate response element-binding protein (CREB) is a transcription factor that promotes tumorigenesis by enhancing cell cycle progression, cell survival, and cellular metabolism [131]. CREB is upregulated in glioma and promotes gliomagenesis. However, understanding of the specific mechanism by which CREB is linked to oncogenic signaling pathways is incomplete. MiR-23a, whose expression was recently found to be transactivated by CREB through its binding to miR-23a regulatory sequences, targets and suppresses PTEN expression, leading to hyperactivation of PI3K-Akt signaling [132].

Studies that identified miRNAs as “gap fillers” in cellular signaling networks suggest that this important class of regulatory molecules, newly identified “fillers” in the “gaps”, not only advance our understanding of the pathological mechanisms of glioma, but also suggest a possible unique significance of miRNAs in the biological basis of the disease. The current knowledge on the biological importance of miRNAs in human glioma is discussed below.

### 3.2. miRNAs Act as “Amplifiers” of Glioma-Associated Cellular Signals

Like other cancer types, glioma development and progression is essentially associated with constitutive overactivation of the oncogenic pathways, which leads to the malignant properties of a tumor cell [9]. In normal cells, regulatory mechanisms typically control the activities of these pathways stringently so that a homeostatic state of the host cell, and the entire cell population in the microenvironment, can be achieved and sustained. The accomplishment of such homeostasis, as shown by many studies, is largely attributed to negative feedback mechanisms that balance the effects of positive stimulators and negative inhibitors of oncogenic molecules on their signaling functions, consequently preventing overactivation of the signaling molecules [133,134,135]. In cancer cells, however, such feedback loops are usually disrupted, amplifying the intensities of oncogenic signals and allowing them to predominate [136,137,138]. In human glioma, miRNAs have been identified as such “amplifier” factors that disrupt the negative feedback loops and cause constitutive activation of the oncogenic signaling molecules.

For example, as a member of the IκB family, IκBα is an intrinsic NF-κB inhibitor yet subject to transactivation by NF-κB, thus forming a negative feedback loop that places NF-κB signaling under strict control and restraining its oncogenic effect [139,140,141]. We recently reported that miR-30e* is highly upregulated in human glioma, can directly target IκBα mRNA and suppress its expression, thereby negating the inhibitory effect of IκBα on NF-κB signaling, leading to the constitutive overactivation of NF-κB. Consequently, the invasiveness and angiogenesis induced is significantly enhanced. Hence, the overexpressed miR-30e* markedly amplifies the stimulation that triggers NF-κB activation [63]. MiR-486 is another NF-κB signaling “amplifier”. MiR-486 simultaneously suppresses the expression of multiple inhibitors of the A20, CYLD and Cezanne proteins. High level of miR-486 expression causes NF-κB overactivation and is associated with the aggressiveness of human gliomas [110]. Identification of these “amplifier” miRNAs provides a new basis for the development of novel anti-glioma strategies.

### 3.3. miRNAs Act as “Fine-Tuners” of Signal Regulation

Under physiological and pathological conditions, signal transduction in a cell is subject to fine-tuning by molecules that buffer noise resulting from different signaling pathways. Such a noise-buffering mechanism is usually associated with the maintenance or dysregulation of cellular functions and phenotype [142,143]. For cells to manage signaling fluctuation, transcriptional regulation of key signaling transducers by proteins has been found to act as a fine-tuning mechanism that buffers noise by reducing the fluctuation. Recent studies show that post-transcriptional regulation by miRNAs might represent a rapid mode of action to fine-tune the signaling network within a cell [130]. For example, the biological functions of the miR-17–92 cluster involve two regulatory loops. In one loop, miR-17–92 represses E2F1 and c-myc, two pro-proliferative transcription factors that are able to reversely activate the transcription of mi-17–92. MiR-17–92 suppresses cell cycle progression and proliferation through this signaling loop. In loop 2, however, miR-17–92 targets CDKN1A, leading to activation of the CCND1/CDK4 complex and cell proliferation. Thus, due to its multi-target property, miR-17–92 provides an intracellular fine-tuning mechanism for the proliferative signal and acts as a noise-buffering mediator in normal cells [40,41,144,145,146,147,148]. However, the miR-17–92 cluster is aberrantly overexpressed during gliomagenesis, promoting the proliferative effects of loop 2, and due to c-myc upregulation, the anti-proliferative function of miR-17–92 in loop 1 is inhibited. Hence, the compromised fine-tuning effect of miR-17–92 enhances the progression of the glioma [39]. The fine-tuning modulation of miRNA in cell signaling is therefore an important subject in the understanding of the pathological attributes of human glioma, and restoring the functions of miRNA fine-tuners holds great potential in glioma treatment.

### 3.4. miRNAs Act as Crosstalk Mediators of Different Signaling Pathways

Cellular signaling pathways are not isolated from one another; instead, crosstalk between and across distinct pathways is common and essential for the functional integrity of the typically complex network [149]. Not only proteins, but also miRNAs, play pivotal roles in signaling crosstalk. A miRNA usually has more than one target, and these targets usually function in different signaling pathways [150]. MiR-21, upregulated in glioma, may contribute to crosstalk among the TGF-β, p53, and EGFR-PI3K-Akt axes. In this context, miR-21 targets Tap63 and several p53 activators, e.g., heterogeneous nuclear ribonucleoprotein K (HNRPK), p53-binding protein 2 (TP53BP2), topoisomerase I-binding (TOPORS), and junction-mediating and regulatory protein (JMY). Moreover, miR-21 can suppress TGF-β receptor II/III, death-associated protein 6 (DAXX, the apoptotic mediator), and TGF-β itself. The suppression of both the TGF-β and p53 pathways leads to the inhibition of apoptosis [43,151]. As mentioned earlier, miR-21 also downregulates the MMP inhibitors RECK and TIMP3 [44]. Thus, miR-21-mediated crosstalk cooperatively links various signaling pathways to the pathogenesis of glioma.

Other examples of the crosstalk-mediating function of miRNA include the miR-221/222 cluster. The cluster directly targets p27, p57, PTEN, TIMP3, p53-upregulated modulator of apoptosis (PUMA), and connexin 43 (CX43), facilitating crosstalk and interaction among multiple signaling pathways associated with cell cycle progression, cell survival/apoptosis, and other essential cellular functions [94,95,152,153]. Hence, by mediating crosstalk among different signal transduction pathways, cooperative regulation of different pathways reinforces the oncogenic or tumor-suppressor roles of miRNAs. Based on these findings, miRNA blocking or overexpression might abrogate more than one signaling cascade and would therefore be therapeutically significant.

## 4. Potential Application of miRNAs in Clinical Management of Glioma: Perspective

Despite recent advances in the understanding of glioma biology and pathogenesis and in the development of new anti-glioma strategies, clinical management of this highly lethal disease still faces serious challenges. First, current glioma diagnosis mostly depends on imageological detection, which usually takes place after the presence of symptoms indicative of late disease become obvious. Early identification of WHO grade I or II gliomas is difficult due to a lack of reliable biomarker(s) and sensitive and specific detection methods. Second, current capacity in the clinic to predict disease prognosis and patient survival is very limited largely due to the clinical and biological heterogeneity of glioma, even those categorized in the same histological type or WHO grade [4]. Better prognostic judgment would require additional parameters. Third, in a similar context, while there has been significant progress in molecular classification of human gliomas for better-guided treatment, e.g., the recently proposed molecular profiling-based subtypes [154] the development of new biomarkers that can distinguish differential glioma patients clinically and support personalized therapy is urgently required. Fourth, the identification of new therapeutic molecular targets to improve glioma treatments with currently limited efficacies, particularly those that can help overcome resistance to conventional chemotherapy and radiotherapy, is needed. Finally, the molecular traits of GSCs, a key basis for developing GSC-targeted therapies, must be understood and validated in clinical glioma. Recent understanding of the roles of miRNAs in glioma development and progression has provided new insights and a practical basis for developing early detection biomarkers, more accurate prognosis prediction tools, and more effective interventional targets.

Numerous miRNAs are associated with the clinical features and outcomes of human glioma (Table 2). For instance, miR-30e*, miR-21, and miR-486 levels in glioma tissues correlate closely with clinical staging and inversely with disease prognosis and patient survival, respectively [45,48,63,110]. The demonstrated stability of miRNA in the peripheral blood, possibly mechanistically due to protection by protein complexes associated with the miRNA molecules, would be noteworthy in the development of practically applicable diagnostic or prognostic biomarkers [155]. By and large, presence of tumor-associated miRNAs in body fluids such as blood and cerebrospinal fluid (CSF), can be detected earlier than in tumor lesions, as pathologic biopsy is mostly performed after typical clinical symptoms appear. Biopsy is particularly hard to carry out for brain tumors due to the difficulty of accessing the primary tumor, making miRNAs an attractive candidate class of biomarkers for early diagnosis of glioma. Furthermore, in addition to the potential value of miRNAs for diagnosis and prognosis, some miRNAs are also complementarily indicative of glioma subtype classification. For example, oligodendrogliomas are sometimes difficult to distinguish from GBM. Genetic alterations, e.g., loss of heterozygosity on chromosomes 1p and 19q, are required quite often to assist pathological diagnosis. A group of miRNAs, including miR-218, miR-21, miR-132, miR-134, miR-155, and miR-409-5p, were reported to be overexpressed by three times as much in GBM compared with oligodendrogliomas, while miR-128 expression in oligodendrogliomas is four times higher than that in GBM. Thus, a specific miRNA signature might aid in the discrimination of oligodendrogliomas from GBM [156]. As the need to establish better detection methodologies for clinical diagnosis, outcome prediction, and molecular classification of gliomas is urgent, the possibility of employing miRNAs as noninvasive diagnostic and prognostic biomarkers warrants further laboratory investigation and clinical evaluation.

**Table 2:** MicroRNA expression in gliomas.

MiRNA	Test	Expression alteration ^a^	Fold change	Relevance to grading ^b^	Relevance to prognosis ^c^	References
Mir-7	qRT-PCR	T(−), C(−)				[28]
MiR-10b	qRT-PCR	T(+), C(+)	90.4 (tissues), 94.8 (U87), 18.4 (U251)	(+)	(−)	[32,33,34]
Mir-15b	MicroRNAarray, qRT-PCR	T(−), C(−)	0.11 (tissues)			[36,37]
Mir-16-1	qRT-PCR	C(−)				[38]
Mir-17	qRT-PCR	T(+), C(+)	2.7–5.6 (tissues)	(+)	(−)	[42]
Mir-17–92 cluster	qRT-PCR	T(+)	1.5–5 (tissues)	(+)		[39]
Mir-21	qRT-PCR, *In situ* hybridization	T(+), C(+)		(+)		[21,45]
Mir-23b	qRT-PCR	T(+), C(+)	7.48–16.04 (cell lines)			[61]
Mir-26a	MicroRNA array	T(+)	6 (GBM tissues)			[62]
Mir-30e*	MicroRNA array, qRT-PCR	T(+), C(+)		(+)	(−)	[63]
Mir-31	qRT-PCR	T(−)				[64]
Mir-34a	qRT-PCR	T(−), C(−)				[157]
Mir-101	qRT-PCR	T(−), C(−)		(−)		[67]
Mir-124	qRT-PCR	T(−), C(−)	57 (GBM tissues)			[23,68]
Mir-125b	Northern blotting, *In situ* localization, MicroRNA array, qRT-PCR	Glioblastoma-associated endothelial cells(−), ATRA-treated human glioma cell lines(−), CD133+ cells(−)				[69,70,71,72]
MiR-125b-2	qRT-PCR	GBM tissues(+), cells treated with TMZ(+), CD133+(+)				[73]
Mir-128	MicroRNA array, qRT-PCR	T(−), C(−)		(−)		[74,75,76]
Mir-137	MicroRNA array, qRT-PCR	T(−), C(−)		(−)		[24]
Mir-145	TCGA analysis, qRT-PCR, MicroRNA array	T(−), C(−)	<0.1 (cell lines)	(−)	(+)	[79,80,81,82]
Mir-146	qRT-PCR	T(−), C(−)				[158,159]
Mir-152	qRT-PCR	T(−), C(−)	0.14 (tissues)			[36]
Mir-153	qRT-PCR	T(−), C(−)				[84]
Mir-181	qRT-PCR	T(−), C(−)		(−)		[86]
Mir-182	qRT-PCR	T(+), C(+)		(+)	(−)	[160]
Mir-184	Stem-loop real-time RT-PCR	T(−)	0.02–0.56 (tissues)			[89]
Mir-195	qRT-PCR	T(−), C(−)	<0.6 (cell lines), <0.4 (tissues)			[91,92]
Mir-204	qRT-PCR	T(−), C(−)			(+)	[93]
Mir-221/222	qRT-PCR, MicroRNA array	T(+), C(+)	2.74 (cell lines), 6.23 (tissues)	(+)	(−)	[101,102]
Mir-296	qRT-PCR	C(+)				[104]
Mir-326	qRT-PCR	T(−)	0.15–0.18 (tissues)	(−)	(+)	[105,106,107]
Mir-410	TCGA analysis, qRT-PCR	T(−),				[108]
Mir-451	qRT-PCR, Fluorescent *in situ* hybridization	T(−), C(−)		(−)		[161,162]
Mir-455	MicroRNA array	Cells resistant to TMZ(+)				[90]
Mir-486	MicroRNA array, qRT-PCR	T(+), C(+)		(+)	(−)	[110]

**^a^** T = Tumor tissues, C = glioma cell lines, (+) = overexpression, (−) = downregulation; **^b^** (+) = Higher WHO grade with higher expression, (−) = higher WHO grade with lower expression; **^c^** (+) = Shorter survival with higher expression, (−) = longer survival with higher expression.

Altered miRNA expression contributes to glioma development and progression, and some of these miRNAs, e.g., miR-21, miR-30e*, and miR-486, might serve as or provide essential regulation to critical nodes in the signaling pathways that are key to glioma pathogenesis. As critical cell signaling nodes are essential mediators that play important roles in a physiological or pathological condition [163], the aforementioned glioma-associated miRNAs might represent a class of potentially effective therapeutic targets. MiRNAs typically modulate protein expression in a timely and efficient fashion via simultaneous suppression of multiple target genes. Moreover, a probable lack of oligonucleotide immunogenicity might provide an additional advantage for the clinical use of miRNAs or their specific antagonists.

MiRNA-based therapy might also be useful for increasing the efficacy of conventional chemotherapy and radiotherapy. As discussed above, some miRNAs, e.g., miR-21 and miR-181a, are associated with acquisition of resistance or sensitization of glioma cells to anti-glioma treatments. Based on these findings, combining miRNA-based therapy and chemotherapy or radiotherapy might lead to higher treatment efficacy. Furthermore, targeting miRNAs that sustain GSC stemness, or employing those suppressing GSC, could be effective in increasing the overall sensitivity of a glioma tumor to chemotherapy and radiotherapy.

Nevertheless, the application of miRNA-based approaches in clinical diagnosis and treatment faces serious challenges. Larger-scale and prospective trials are needed to validate the effectiveness of miRNAs that were identified in studies using relatively limited patient samples in disease diagnosis, staging, and molecular subtyping, or to predict disease progression, patient survival, and efficacy of therapy. Moreover, the methods currently used to quantify miRNAs in surgical specimens and to correlate them qualitatively or semi-qualitatively to the clinical features of a glioma case should be improved for practical application. Defining cut-off values that can effectively distinguish “high” and “low” expression of a particular miRNA and avoid interference from nonspecific detection of other miRNAs with high sequence similarities is always a challenge. Furthermore, miRNA detection should be explored in clinically accessible samples, e.g., blood or CSF, which also offers opportunities to detect glioma-associated miRNAs at an early disease stage or before symptoms occur. In addition, miRNA-based functional imaging of clinical glioma could be an interesting direction to investigate [164,165].

Development of miRNA-based anti-glioma treatments presents even more challenges than those against other cancer types. Issues remaining to be addressed include specific and effective systemic delivery of miRNA and miRNA-targeted agents into the CNS protected by the blood-brain barrier, and prevention of off-target effects of a miRNA or anti-miRNA construct. There have been efforts to facilitate the delivery of miRNAs or their inhibitors *in vivo*. The efficacy of cholesterol-conjugated antisense oligonucleotides against oncomirs and poly(amido-amine)-carried tumor-suppressive miRNA oligonucleotides has been tested against xenografted glioma in animal models with fairly positive results. With encouraging results from recent human studies using synthetic RNAi as anti-tumor therapeutic agents [166,167], future studies investigating the efficiency, efficacy, and safety of miRNA-based therapeutic strategies are warranted.

## 5. Conclusions

microRNAs have been suggested by a large number of studies to play a pivotal role in the development of malignant phenotype of glioma, characterized by enhanced cell survival, proliferation, tumor angiogenesis, dedifferentiation as well as generation of cell stemness. Discoveries of the biological significance of miRNAs dysregulation in glioma cells not only fill some disconnected gaps between previously identified, but disconnected, mechanistic components underlying the pathogenesis of the disease, but also provide a model system through which the role of miRNA in tumorigenesis and cancer progression can be better understood. Moreover, it has been increasingly revealed that changes in the expression level of particular miRNAs might represent a new class of benchmarks indicative of the presence and/or progression of glioma tumors, and therefore might be of diagnostic or prognostic value. Furthermore, it is important to also acknowledge that as key molecules involved in mediating cellular behaviors essential for establishment of primary tumors as well as invasive growth of glioma, miRNAs are potentially promising targets of future anti-glioma intervention, despite the apparently pressing challenges lying ahead along the path towards the eventual clinical application of miRNA-based therapies. Thus, further and more in-depth mechanistic studies on the biological basis upon which miRNAs contribute to gliomagenesis and lethality of the disease, as well as translational research to overcome the technical barriers impeding the applicability of miRNA as diagnostic, prognostic, therapeutic or preventive tool, are equally urgent. 
